# Merits of Surgical Comanagement of Patients With Hip Fracture by Dedicated Orthopaedic Hospitalists

**DOI:** 10.5435/JAAOSGlobal-D-20-00231

**Published:** 2021-03-10

**Authors:** Nidhi Rohatgi, Yingjie Weng, Jessie Kittle, Neera Ahuja

**Affiliations:** From the Division of Hospital Medicine, Department of Medicine, Stanford University School of Medicine, CA (Dr. Rohatgi, Dr. Kittle, and Dr. Ahuja), and the Quantitative Sciences Unit, Division of Biomedical Informatics Research, Department of Medicine, Stanford University School of Medicine, CA (Mr. Weng).

## Abstract

Rotating medical consultants, hospitalists or geriatricians, are involved in the care of patients with hip fracture, often after medical complications have already occurred. In August 2012, we implemented a unique surgical comanagement (SCM) model in which the same Internal Medicine hospitalists are dedicated year-round to the orthopaedic surgery service. We examine whether this SCM model was associated with a decrease in medical complications, length of stay, and inpatient mortality in patients with hip fracture admitted at our institution, compared with the previous model.

**Methods:**

We included 2,252 admissions to the orthopaedic surgery service with a hip fracture between 2009 and 2018 (757 pre-SCM and 1495 post-SCM). We adjusted for age, Charlson comorbidity score, and operating time in all regression analyses.

**Results:**

Mean Charlson comorbidity score (1.6 versus 1.2) and median case mix index (2.1 versus 1.9) were higher in the post-SCM group. A 32% decrease was observed in the odds of having ≥1 medical complication(s) (odds ratio, 0.68 [95% confidence interval, 0.50 to 0.91], *P* = 0.009) post-SCM. No change was observed in length of stay or inpatient mortality despite an increase in medical complexity post-SCM.

**Conclusion:**

Having dedicated orthopaedic hospitalists may contribute to fewer medical complications in patients with hip fracture.

The annual number of hip fractures in the United States is estimated to reach 289,000 by 2030.^1,2^ Most patients with hip fractures are ≥65 years of age,^3^ and ≥85% of these patients have notable medical comorbidities.^4^ Over a third of these patients have postoperative medical complications (distinct from surgical complications); these medical complications predict mortality after hip fracture.^4–6^

Consequently, comanagement models of care have been popularized in which geriatricians^7,8,9,10,11,12^ or hospitalists^13,14,15,16,17,18^ participate in the care of patients with hip fracture during their hospital stay. However, in many of these models, the hospitalist rotating on the General Medicine consult service on that day would be consulted “as needed” by the orthopaedic surgery service (often after medical complications had already occurred). In some of these models, geriatricians would proactively follow these patients, but not on the weekends or after hours.

In August 2012, we implemented a surgical comanagement (SCM) model on the orthopaedic surgery service at our institution.^17,18^ Our SCM model is unique because the same Internal Medicine hospitalists are dedicated year-round to the orthopaedic surgery service. These hospitalists do not see patients on the General Medicine service. The SCM hospitalists build a rapport with the surgical team and develop a skillset in managing these surgical patients from admission to discharge and also facilitate care coordination with the outpatient providers at the time of discharge. In this study, we examine whether this SCM model was associated with a decrease in medical complications, length of stay (LOS), and inpatient mortality in patients with hip fracture admitted at our institution, compared with the previous model in which rotating hospitalists on the General Medicine consult service would be occasionally consulted on these patients.

## Methods

### Design, Setting, and Patients

This is a pre-post study comprising 2,252 admissions for hip fracture at our 477-bed academic medical center. The average volume of admissions for hip fracture on the orthopaedic surgery service at our institution is 255 patients per year; the daily census is approximately 50 patients. We included admissions to the orthopaedic surgery service for displaced and nondisplaced osteoporotic, stress, or pathological femur and acetabular native hip fractures. These were identified using International Classification of Diseases (ICD) codes. We excluded periprosthetic fractures and admissions for hip fracture to services other than orthopaedic surgery (e.g., General Medicine, Cardiology, and General Surgery/Trauma).

We defined the preintervention (or pre-SCM) group as all admissions for hip fracture to the orthopaedic surgery service between January 1, 2009, and July 31, 2012. We defined the postintervention (or, post-SCM) group as all admissions with hip fracture to the orthopaedic surgery service between September 1, 2012, and June 30, 2018. We excluded admissions for hip fracture in August 2012 because we transitioned to the SCM model. Data were abstracted from electronic medical records. Our Institutional Review Board exempted this study from further review.

### Surgical Comanagement Workflow

The structure and workflow of SCM is detailed in previous articles.^17,18^ On weekdays between 8 am and 5 pm, two SCM hospitalists staff the orthopaedic surgery service. One SCM hospitalist takes after-hour calls every weeknight and sees patients on the weekend. All patients with hip fracture admitted to the orthopaedic surgery service are seen by SCM hospitalists for preoperative risk assessment, medical optimization when possible, and postoperative medical care. SCM hospitalists done a comprehensive history and physical examination and document the patient's perioperative medical management plan, including strategies for preventing medical complications.^19^ Because SCM hospitalists enter orders on the electronic health records (instead of simply giving verbal recommendations or leaving recommendations in the patient notes), most of their recommendations are implemented by the orthopaedic surgery service. Given over three-quarters of the geriatric patients may not have the correct medication list documented,^20^ SCM hospitalists contact outpatient providers and/or caregivers to obtain the correct medication list and/or medical histories. Any preoperative workup or imaging ordered by SCM hospitalists (e.g., transthoracic echocardiogram) is done in accordance with evidence-based guidelines.^21^ SCM hospitalists are also available on the patient units to provide timely intervention in case of medical deterioration although the surgical team may be in the operating room.^22^

### Interventions by Surgical Comanagement

SCM hospitalists and the orthopaedic surgery service worked together on the admission and postoperative order set for patients admitted with hip fracture. Protocols for prevention of acute kidney injury and delirium were implemented.^23,24^ A hip fracture pathway was implemented in collaboration with the emergency department where the SCM hospitalist would promptly see any patient admitted with hip fracture before 5 pm for perioperative risk assessment or staff the patient by phone with the medicine house staff who would see the patient after-hours. In addition to establishing new protocols, examples of daily interventions by SCM hospitalists include careful management of fluid balance, blood pressure, anticoagulation, analgesia, and titration of cardiac medications. New derangements such as orthostatic hypotension, hyponatremia, infections, venous thromboembolism, chest pain, arrhythmia, hypoxia, urinary retention, nausea, or constipation are promptly evaluated and treated. SCM hospitalists participate in goals of care discussions, answer any medical questions the patient/caregivers may have, and coordinate medical care between the inpatient team and outpatient providers.

### Outcomes

#### Primary Outcome

The primary outcome was the proportion of admissions with ≥1 of the following medical complications: sepsis, pneumonia, urinary tract infections, delirium, acute kidney injury, atrial fibrillation/flutter, or ileus. These were diagnosed and documented by any clinician involved in the care of the patient based on their clinical judgment. We included these medical complications because they can be directly affected by a hospitalist on a surgical service. We defined all medical diagnoses using ICD-9 or ICD-10 codes (Supplemental Table 1, Supplemental Digital Content 1, http://links.lww.com/JG9/A116). A diagnosis was defined as a “medical complication” if it was coded as “not present on admission.”

#### Secondary Outcomes

Our secondary outcomes were LOS and inpatient mortality during the index admission.

### Statistical Analysis

Patient demographics and clinical characteristics were compared between pre-SCM and post-SCM, and standardized mean differences were calculated for each variable using the method implemented in the “tableone” package in R.^25^ Standardized mean difference is a measure of effect size and defined as the mean difference between the two groups (pre-SCM and post-SCM) over the pooled standard deviation. Charlson comorbidity score, which represents the summated weight assigned to a number of comorbidities, was calculated using the “icd” package in R using the Quan-Deyo method.^26^

We used logistic regression with logit link to assess the difference between pre-SCM and post-SCM for our two binary outcomes (i.e., proportion of patients with ≥1 medical complication and inpatient mortality) and reported odds ratios (ORs). Gamma regression with identity link was done for our continuous outcome (i.e., LOS), and relative risk was reported. Beta coefficient was reported to estimate the difference in LOS between pre-SCM and post-SCM under their original scales. We adjusted for age, Charlson comorbidity score, and operating time in all regression analyses. SAS version 9.4 and R version 3.6.3 were used for all analyses.

## Results

A total of 2,252 admissions for hip fracture to the orthopaedic surgery service were included in the analysis: 757 in the pre-SCM group and 1,495 in the post-SCM group. Patient characteristics are shown in Table 1. Patients in the post-SCM group had a higher mean Charlson comorbidity score (1.6 versus 1.2), higher median case mix index (2.1 versus 1.9), and shorter median operating time (109 minutes versus 123 minutes). Most of the admissions were from home in both the groups. Patients in the post-SCM group were more likely to be discharged to a rehabilitation facility (59.2% versus 48.7%). Among patients admitted from home, there were more discharges to rehabilitation facility in the post-SCM group (50.2% versus 35.4%).

**Table 1:** Patient Characteristics in the Presurgical and Postsurgical Comanagement Groups

Characteristic	Pre-SCM (n = 757) (January 2009 to July 2012)	Post-SCM (n = 1495) (September 2012 to June 2018)	SMD
Age, median (IQR), yr	72 (55-85)	75 (59-87)	0.14
Male, No. (%)^a^	303 (40.0)	622 (41.6)	0.03
Race/ethnicity, No. (%)			0.14
White	585 (78.1)	1099 (74.6)	
Married/partnered, No. (%)	336 (44.4)	700 (47.3)	0.06
Annual income, USD, median (IQR)^b^	102,500 (71,839-12,736)	104,481 (80,392-12,736)	0.007
Primary insurance, No. (%)			0.14
Medicare	378 (49.9)	853 (57.1)	
Commercial/self-pay	342 (45.2)	574 (38.4)	
Medi-Cal	37 (4.9)	68 (4.5)	
Case mix index, median (IQR)	1.9 (1.5-2.1)	2.1 (1.9-2.1)	0.24
Case mix index > 2.5, No. (%)	312 (41.2)	836 (55.9)	0.29
Charlson comorbidity score, mean (SD)	1.2 (1.7)	1.6 (2.1)	0.20
Medical comorbidities, No. (%)			
Dementia	31 (4.1)	158 (10.6)	0.25
Congestive heart failure	69 (9.1)	191 (12.8)	0.12
Chronic lung disease	107 (14.1)	282 (18.9)	0.13
Diabetes mellitus	112 (14.8)	217 (14.5)	0.008
Malignancy	67 (8.9)	181 (12.1)	0.10
Stroke	31 (4.0)	43 (2.9)	0.06
Renal disease	108 (14.3)	295 (19.7)	0.15
Liver disease	24 (3.2)	56 (3.7)	0.03
General anesthesia, No. (%)	742 (98.4)	1465 (98.4)	0.002
Cut-to-close operating time, median (IQR)	123 (89-179)	109 (74-161)	0.22
Admit source, No. (%)			
Home	585 (77.5)	1190 (85.1)	0.19
Rehabilitation facility	17 (2.3)	32 (2.3)	0.002
Outside hospital	65 (8.6)	176 (12.6)	0.13
Other	88 (11.7)	1 (0.1)	0.51
Discharge destination, No. (%)			
Home	308 (40.7)	512 (34.4)	0.13
Rehabilitation facility	369 (48.7)	882 (59.2)	0.21
Other	80 (10.6)	96 (6.4)	0.15
Admit source to discharge destination, No. (%)			
Home to home	262 (34.7)	434 (31.1)	0.07
Home to rehabilitation facility	267 (35.4)	700 (50.2)	0.30
Rehabilitation facility to rehabilitation facility	11 (1.5)	25 (1.8)	0.03

IQR = interquartile range, SCM = surgical comanagement, SMD = standardized mean difference, USD = United States dollar

a Missing values: race/ethnicity, 8 in pre-SCM and 21 in post-SCM; marital status, 1 in pre-SCM and 15 in post-SCM; income, 12 in pre-SCM and 33 in post-SCM; case mix index, 2 in post-SCM; anesthesia type, 4 in pre-SCM and 9 in post-SCM; operating time, 4 in pre-SCM and 8 in post-SCM; admit source, 2 in pre-SCM and 96 in post-SCM; discharge destination, 5 in post-SCM; admit source to discharge destination, 2 in pre-SCM and 101 in post-SCM.

b Median zip code level household income from 2010 United States Census.

**Figure 1:**
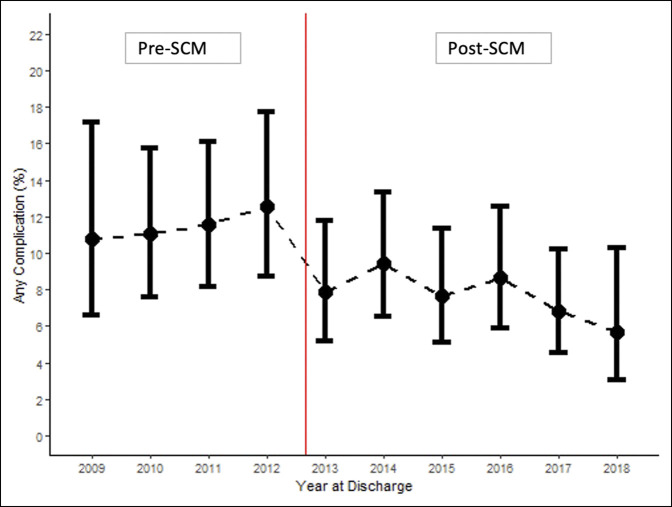
Graph showing the adjusted rate of ≥1 medical complication(s) by year before and after the implementation of SCM. The red line represents when SCM was implemented in August 2012. SCM = surgical comanagement

Table 2 shows the primary and secondary outcomes in the pre-SCM and post-SCM groups. A 32% decrease was observed in the odds of having ≥1 medical complication(s) (OR, 0.68 [95% confidence interval (CI), 0.50 to 0.91], *P* = 0.009) post-SCM (Figure 1). Among the medical complications, a significant decrease was observed in the odds of having acute kidney injury (OR, 0.57 [95% CI, 0.36 to 0.90], *P* = 0.016) and ileus (OR, 0.43 [95% CI, 0.19 to 0.99], *P* = 0.046). A decrease was observed in the odds of all other medical complications, but the difference was not statistically significant. No notable change was observed in LOS or inpatient mortality.

**Table 2:** Primary and Secondary Outcomes

Outcome	Unadjusted Analysis			Adjusted Analysis^a^	
	Pre-SCM	Post-SCM	SMD	Estimate	*P*
≥1 Medical complication(s)	n = 95 (12.5%)	n = 156 (10.4%)	0.07	OR 0.68 [95% CI, 0.50 to 0.91]	0.009
LOS (d)	Median = 4.2 (IQR: 3.1-5.6)	Median = 3.8 (IQR: 3.0-5.5)	0.03	RR −0.08 [95% CI, −0.31 to 0.15]	0.487
Inpatient mortality	n = 14 (1.8%)	n = 21 (1.4%)	0.04	OR 0.52 [95% CI, 0.25 to 1.06]	0.073

CI = confidence interval, IQR = interquartile range, LOS = length of stay, OR = odds ratio, RR = relative risk, SCM = surgical comanagement, SMD = standardized mean difference

a Adjusted for age, Charlson comorbidity score, and operating time.

## Discussion

In our study, we report a 32% decrease in medical complications among patients admitted with hip fracture after the implementation of SCM, with no change in LOS or inpatient mortality. We believe that by having the same hospitalists dedicated to the orthopaedic surgery service year-round, these SCM hospitalists have developed a unique skillset in managing patients with hip fracture. Although the post-SCM group had more comorbidities, no increase was observed in LOS or inpatient mortality compared with pre-SCM. The higher Charlson comorbidity score in the post-SCM group compared with pre-SCM could have been due to patient selection by surgeons or better documentation of comorbidities by SCM hospitalists.

### Current Literature on Comanagement Models of Care

Previous studies have assessed the impact of having hospitalists or geriatricians participate in the care of patients with hip fracture during their hospital stay. Some of these studies reported a decrease in the 7-day readmission rate,^13^ LOS,^12,14–16^ preoperative echocardiograms,^8,14^ time to surgery,^8,10,12,14^ inpatient mortality,^8,9^ 1-year mortality,^11,12^ pressure ulcers,^10^ prescription of potentially inappropriate medications,^27^ and cost of care.^14^ Several of the studies reported an improvement in 4-month mobility among home-dwellers,^7^ better pain control,^8^ and an increase in assessments for osteoporosis.^10,14,16^ On the other hand, some studies reported no difference in the 30-day readmission rate,^8,14,15^ LOS,^8,28^ time to surgery,^15^ 30-day mortality,^14^ falls,^10^ or prescription of osteoporosis medications.^8^ One study that used data from the National Surgical Quality Improvement Program showed that comanagement models were associated with an increase in 30-day mortality and morbidity.^28^

However, it is unclear whether the “comanagement” models reported in these studies above were in actuality similar to our pre-SCM model, where rotating hospitalists on the medicine consult service or geriatricians would be consulted (often after the medical complication had occurred) or where geriatricians would follow these patients during the daytime on weekdays but having medicine consult service see these patients after-hours and on weekends. We believe, in a true SCM model, hospitalists or geriatricians should (1) proactively follow patients with hip fracture throughout their hospital stay with 24-hour coverage to be able to effectively prevent and/or manage medical complications, (2) develop an understanding of the surgical course of these patients and the surgeon's viewpoint, (3) be familiar with the literature in perioperative medicine, and (4) have a rapport with the surgical team to actually influence decisions and outcomes.

In our study, we included the outcomes that could be affected by hospitalists on the orthopaedic surgery service at our institution. Although included in previous studies, it is unclear whether geriatricians or hospitalists who see patients with hip fracture during the hospital stay can actually affect longer-term outcomes such as 4-month mobility or 1-year mortality rates or even 30-day readmission rates or not. Several studies reported time to surgery as one of their outcomes; however, this may be affected by the availability of the operating room or the surgeon or the surgical supplies, and not necessarily by delay in preoperative risk assessment by medical consultants or comanagers. In the HIP ATTACK trial that analyzed accelerated surgery for hip fracture within 6 hours compared with the standard of care, 26.2% of the patients who were eligible for the trial could not be enrolled because the operating room or the surgeon were not available.^29^ Variables that may contribute markedly to the LOS but cannot be affected by SCM often include the following: (1) delays related to the availability of the operating room, the surgeon, the emergency department workflow, or diagnostics; (2) patients who may have recently taken an anticoagulant requiring the surgical team to postpone the surgery; or (3) challenges in coordinating discharge to rehabilitation facilities because of insurance or bed availability or patient/caregiver preferences.

### Cost-Effectiveness of the Surgical Comanagement Model

Although implementation of SCM can be perceived to be cost-prohibitive, it makes fiscal sense.^17,18,30–32^ A recent study done an economic analysis of having dedicated hospitalists comanage patients ≥80 years of age admitted with osteoporotic hip fracture. This study showed that the SCM model of care was cost-effective for hospitals with ≥54 cases per year and resulted in cost savings for hospitals with ≥300 cases per year.^31^

By contrast, another study reported that SCM by hospitalists for patients with joint arthroplasty may increase the total cost of care, driven by the increased cost of imaging and laboratory services, although the total LOS may decrease and contribute to notable cost reduction.^33,34^ By adhering to evidence-based guidelines, using order sets, and avoiding daily routine laboratory and imaging, these costs can be reduced. Reduction in medical complications after implementation of SCM can be considered an inherent cost-savings benefit as well. With an increase in the capture rate of medical diagnoses in their notes, SCM hospitalists can also contribute to improved observed to expected ratios and overall hospital rankings and increase reimbursement to the hospitals by allocation of patients to their appropriate Medicare Severity Diagnosis-Related Group.^34^

### Limitations and Strengths

This study has several limitations. First, this is a single-center retrospective study at an academic medical center. Second, we did not have a control group, and multiple hospital-wide interventions or improvements in surgical or anesthetic techniques over time may have affected our outcomes. Third, this is an observational study where unobserved variables may bias the results. Fourth, we used ICD codes to identify medical complications that rely on the quality of physician documentation. Fifth, we did not have data on longer-term medical outcomes after discharge from the hospital or the impact of improved medical outcomes on functional recovery. Finally, we did not have financial data to assess the cost-effectiveness of this model in this group of patients. This study has several strengths. First, we report the impact of a unique SCM model. Second, this is a large study assessing the impact of SCM model in patients with hip fracture that spans over 9.5 years. Third, we noted a decrease in medical complications in the post-SCM group although the quality of documentation may have only improved in that period.

## Conclusion

A 32% decrease was observed in medical complications among patients admitted with hip fracture after the implementation of SCM despite an increase in medical complexity. Our unique SCM model where we have the same hospitalists dedicated to the orthopedic surgery service year-round may have contributed to the decrease in medical complications.
